# Resting-state theta/beta EEG ratio is associated with reward- and punishment-related reversal learning

**DOI:** 10.3758/s13415-017-0510-3

**Published:** 2017-06-05

**Authors:** Iris Schutte, J. Leon Kenemans, Dennis J. L. G. Schutter

**Affiliations:** 1 Department of Experimental Psychology and Psychopharmacology, Helmholtz Institute, Utrecht University, Heidelberglaan 1, 3584 CS Utrecht, The Netherlands; 2 Donders Institute for Brain, Cognition and Behaviour, Radboud University Nijmegen, Nijmegen, The Netherlands

**Keywords:** Electroencephalogram, Beta oscillations, Punishment, Reversal learning, Reward, Theta oscillations

## Abstract

Prior research has shown that the ratio between resting-state theta (4–7 Hz)-beta (13–30 Hz) oscillations in the electroencephalogram (EEG) is associated with reward- and punishment-related feedback learning and risky decision making. However, it remains unclear whether the theta/beta EEG ratio is also an electrophysiological index for poorer behavioral adaptation when reward and punishment contingencies change over time. The aim of the present study was to investigate whether resting-state theta (4–7 Hz)-beta (13–30 Hz) EEG ratio correlated with reversal learning. A 4-min resting-state EEG was recorded and a gambling task with changing reward-punishment contingencies was administered in 128 healthy volunteers. Results showed an inverse relationship between theta/beta EEG ratio and reversal learning. Our findings replicate and extend previous findings by showing that higher midfrontal theta/beta EEG ratios are associated with poorer reversal learning and behavioral adaptive responses under changing environmental demands.

## Introduction

The sensitivity to reward and punishment signals guides decision-making by exploiting acquired knowledge to shape a long-term adaptive strategy (Bechara, Damasio, Damasio, & Anderson, 1994). Prior research has demonstrated that low punishment sensitivity together with a strong reward dependency predicts risky disadvantageous decision making, whereas high punishment sensitivity and weak reward dependency predicts advantageous decision making (van Honk, Hermans, Putman, Montagne, & Schutter, 2002).

There is now ample evidence that spontaneous oscillations play a critical role in brain functions (Fries, 2005; Knyazev, 2007) and electrophysiological studies have demonstrated that the sensitivity to reward and punishment is reflected in spontaneous oscillatory activity (Schutter & van Honk, 2005; Schutter, de Weijer, Meuwese, Morgan,. & van Honk, 2008). Specifically, we previously showed a positive association between the ratio of relatively slow theta oscillations (4–7 Hz) to beta oscillations (13–30 Hz) and disadvantageous decision making during the (Iowa) gambling task (Schutter & Van Honk, 2005; Massar, Kenemans & Schutter, 2014). During the Iowa Gambing Task (IGT) disadvantageous decisions are associated with large immediate rewards, but in the long run these decisions result in even larger punishments, whereas advantageous decisions are linked to moderate immediate rewards but smaller punishment. It was proposed that the theta/beta EEG ratio is the manifestation of a brain state that promotes reward-drive. However, as indicated, another feature of the IGT as traditionally implemented is that involves a clear reversal aspect, that is, choices that are initially advantageous suddenly become mainly disadvantageous. It is possible that theta/beta EEG ratio reflects a relative inability to adapt to such reversals, rather than reward sensitivity. In the present work we explicitly address this possibility. In the following, we first provide an overview of functional connotations of theta and beta activity separately; after that we provide an integrated perspective featuring the theta/beta EEG ratio.

Spontaneous slow oscillations in the theta range (4–7 Hz) have been characterized neuro-anatomically and -physiologically. Scheeringa et al. (2008) reported a negative correlation between theta power and BOLD responses in medial-frontal cortex, as well as in a number of other cortical regions. One interpretation suggested by the authors is a general increase in low-frequency EEG power (including theta power) with decreasing BOLD signals across the cortex. While this relation between theta activity and BOLD may reflect biophysical rather than functional aspects, it is strongest in medial-frontal regions. An MFC-based generator was also confirmed by source-localization analysis (Scheeringa et al., 2008).

A large body of work suggests that mid-frontally generated theta activity is linked to activity of the anterior cingulate cortex (ACC), in line with the findings by Scheeringa et al. (2008). Task-related theta activity has been assessed mainly in response to or during stimuli that induce conflict or uncertainty (e.g., Cavanagh & Frank, 2014; Cavanagh & Shackman, 2015; Cavanagh, Zambrano-Vazquez, & Allen, 2012; van Driel, Swart, Egner, Ridderinkhof, & Cohen, 2015; Van de Vijver, Ridderinkhof, & Cohen, 2011). A recent study applying transcranial alternating current stimulation (tACS) suggests a causal relationship, as tACS at theta frequency reduced the performance manifestation of conflict (van Driel, Sligte, Linders, Elport, & Cohen, 2015). Conflict-induced theta activity is generally thought to reflect an error signal carried by disinhibition of medial-frontal-cortex neurons (Cohen, 2014). Such error signals would especially occur when things get more difficult than average or than expected.

Spontaneous theta activity is thought to have a similar error-like MFC disinhibition origin. Another, related perspective is that theta activity is evoked by feedback signals. It scales proportionally to the valence (i.e., stronger for negative than for positive feedback), to the size of the prediction error (i.e., stronger with larger differences between expected and obtained reward or punishment), and also with the learning rate within a task (Mas-Herrero & Marco-Pallarés, 2014).

As noted by Cohen (2014), conflict-related theta activity is a temporary non-phase-locked increase (“burst”) in endogenous-theta power, triggered by conflict-detecting neural units in deeper layers of the MFC. Endogenous theta activity is spontaneous and thought to be generated in more superficial layers that also receive inputs from subcortical reward and punishment structures. From this perspective, enhanced spontaneous theta activity would reflect a condition of less reward than expected, even in the absence of discrete signals (such as feedback stimuli) of obtained reward magnitude. As such it could reflect a relatively enhanced continuous striving to obtain rewards, even in the absence of task-related stimuli that signal the possibility and the subsequent obtainment (or not) of reward. Note that while scalp-recorded theta activity may be driven by subcortical inputs, it is mainly or exclusively a direct reflection of cortical activity.

Beta (13–30 Hz) activity is a type of fast oscillatory activity associated with top down control and decision-making processes (Donner & Siegel, 2011). Increasing evidence indicates that beta oscillatory activity reflects active inhibitory processes involved in maintenance of the current motor and cognitive state (Engel & Fries, 2010). A recent review (Marco-Pallarés, Münte, & Rodríguez-Fornells, 2015) emphasizes the beta response to rewarding events which is “in charge of transmitting a fast motivational signal to downstream brain structures” (p. 4; see also Van den Vijver et al, 2011).

Given these presumed complementary associations between theta versus beta oscillations on the one hand, and reward sensitivity versus reward processing on the other, it seems natural to evaluate theta power relative to beta power. A conceptual underpinning is that theta activity is in part driven by subcortical signals, whereas beta activity represents endogenous cortical activity (Schutter & van Honk, 2005; Schutter, Leitner, Kenemans, & van Honk, 2006). Hence, the theta/beta EEG ratio reflects the inverse of cortical regulating signals relative to subcortical to-be-regulated activity. Given that spontaneous theta activity reflects uncertain anticipation of reward, the spontaneous beta rhythm could represent a quenching signal towards subcortical structures that drive the theta activity, signaling that reward anticipation can be toned down in average everyday-life or laboratory conditions. Beta oscillations in this perspective are most prominent across anterior midline sites. They should be distinguished from more lateral beta oscillations that have been associated with error-related sensory-motor adjustments (Luft, Takase, & Bhattacharya, 2014).

In the natural world reward-punishment contingencies are subject to change and as a result individuals may encounter situations with different pay-off schedules. It is critical for the individual to react to such changes in reward-punishment contingencies by rapidly shifting to situationally appropriate decision-making strategies (Clark, Cools, & Robbins, 2004). Such an adaptation in the situation in which reward-punishment contingencies reverse has been termed reversal learning (Bechara, Damasio, Tranel, & Damasio, 2005). Reversal learning requires that an individual makes an optimal trade-off between exploiting acquired knowledge and exploring other response options that may lead to more profit (Daw, O'Doherty, Dayan, Seymour, & Dolan, 2006). Functional neuroimaging research has found that the subcortical reward circuit mediates exploitation in concert with the medial frontal cortex, whereas exploratory decision-making in uncertain environments involves activation of the frontopolar cortex (Daw et al., 2006).

In sum, task-related theta activity might signal the need to adjust the level of cognitive control to optimize behavior during uncertainty, conflict or punishment (anxiety-provoking situations) (Cavanagh & Frank, 2014; Cavanagh & Shackman, 2015). This is especially manifest in the association between theta activity and aspects of reversal learning (Mas-Herrero & Marco-Pallarés, 2014), whereas beta activity may represent a quenching signal towards subcortical structures that drive the theta activity. Building on our earlier work (Schutter & Van Honk, 2005; Massar et al., 2014; see above), we ask whether an association exists between endogenous theta/beta EEG ratio and aspects of reversal learning. We previously reported a positive association between theta/beta EEG ratio and risky decisions. The latter could be related to a negative association with the ability to adapt to changing choice-reward/punishment contingencies. Therefore the present hypothesis is that higher theta/beta EEG ratios are associated with poorer reversal learning. In addition, we explored the relations between self-reported sensitivity to reward and punishment and reversal learning. As prior research has shown that low punishment sensitivity and strong reward dependency are associated with disadvantageous decision-making (van Honk et al., 2002), we anticipated that high reward sensitivity and low punishment sensitivity would be associated with poorer reversal learning.

## Methods

### Participants

One hundred and thirty-three volunteers participated in the study. Participants were recruited through advertisement at the campus of Utrecht University. Five subjects were excluded because of prior experience with the task. The final sample consisted of 128 participants (mean age: 22.3 years (SD: 3.3 years); 87 females; 122 right-handed). All were unaware of the aim of the experiment and had no prior experience with the task. All subjects were healthy and none of them had a history of psychiatric or neurological conditions and none of them used psychoactive medication. All subjects had normal or corrected-to-normal vision. Participants were requested to abstain from caffeine and smoking on the day of testing. Subjects gave written informed consent and were paid for participation or received study credits instead. The study was approved by the local ethics committee of the faculty of Social and Behavioral Sciences of Utrecht University.

### The reversal learning gambling task

**Fig. 1:**
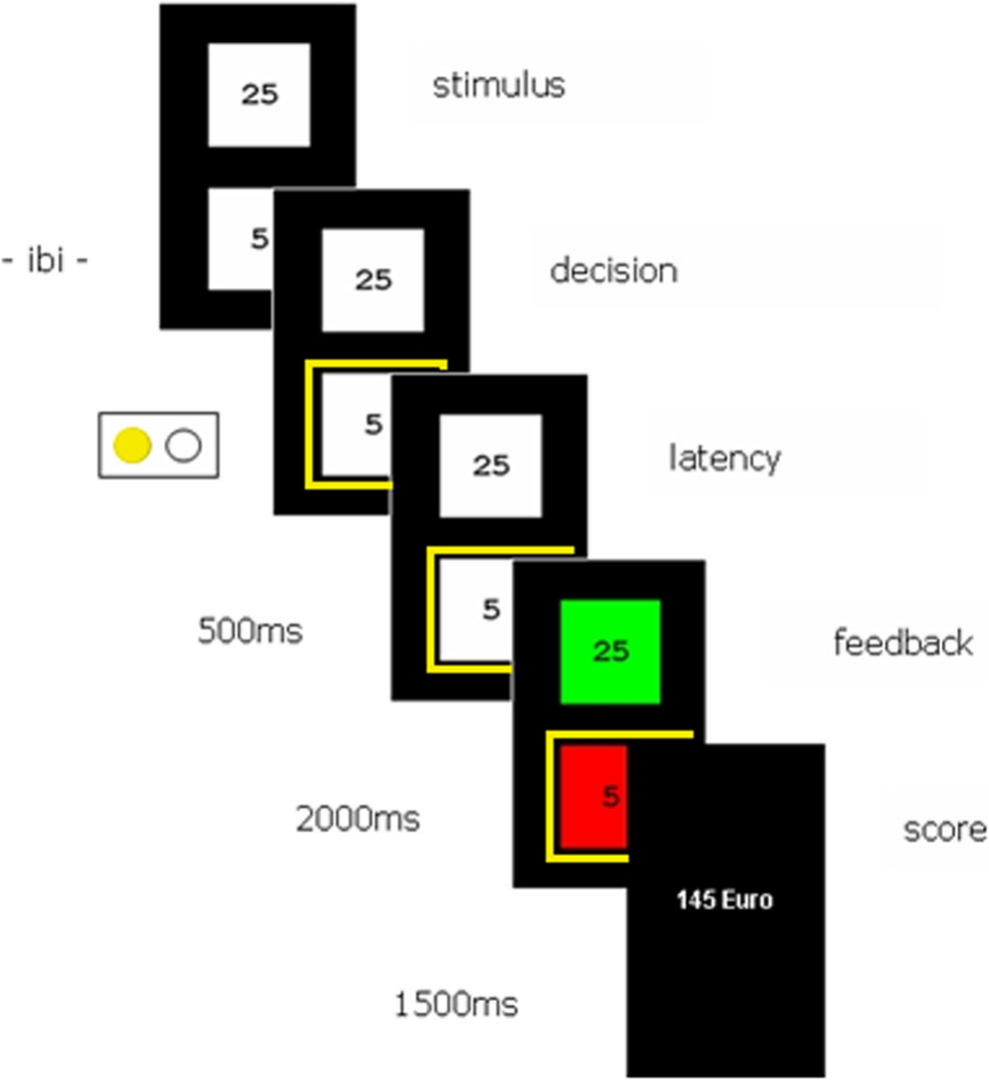
Typical trial sequence. During the reversal learning gambling task participants either choose a high or low amount. After a 500-ms delay feedback is shown whether the amount has been won (the chosen value turns green) or lost (the chosen value turns red). The amount that has not been chosen also turns red or green to provide additional feedback. Finally, the total score so far is shown. The yellow rectangles indicate the participant’s choice. Note that these rectangles are only displayed in this figure for illustrational purposes and are not displayed during the actual task. In this particular example, the participant has chosen the low amount (left button-press) and has lost

The task is based on the Iowa gambling task (Bechara et al., 1994) and the affective reversal learning task by Fellows and Farah (2003). On each trial participants chose one of two squares that contained an amount of money that could either be won or lost. The aim was to win as much fictitious money as possible. On each trial a combination of a high and low value was presented. Participants could either engage in high-risk decision making by choosing the high monetary value or in low-risk decision making by choosing the low monetary value. Eight possible stimulus combinations, namely [5-25], [25-5], [10-30], [30-10], [15-35], [35-15], [20-40], and [40-20] were used and presented vertically and in random order. Participants made a high risk choice by pressing the right mouse button and a low risk choice by pressing the left mouse button. For instance, when presented with [25-5] or [5-25], participants would take high risk by choosing the numeral 25 (i.e., press the right button) over 5 (i.e., pressing the left button). Feedback was provided 500 ms after the subject’s decision by coloring the squares either green or red. The amount of fictitious money displayed in the square was either won or lost depending on whether the chosen square turned green (won) or red (lost). The non-chosen square also colored green or red to provide additional feedback on what would have been the outcome of their alternative choice. Each trial was ended by providing a balance update (i.e., score) that was displayed 2,000 ms after feedback onset for a duration of 1,500 ms. The inter-trial onset time varied between 800 and 1,200 ms. Figure 1 displays the events sequence of a typical trial. The gambling task consisted of two practice trials and six rounds of 20 trials each and was divided in three phases with a different reward-punishment (R-P) schedule for high-risk decision making. During phase 1 (i.e., round 1 and 2, trial 1–40) choosing the high amount (risk-taking) was rewarded in 80% of the trials. During phase 2 (i.e., round 3 and 4, trial 41–80) the reward-punishment schedule was reversed and choosing the high amount was only rewarded in 20% of the trials (choosing the low amount was rewarded in 80% of the trials). During phase 3 (i.e., round 5 and 6, trial 81–120) choosing the high amount (risk-taking) was again rewarded in 80% of the trials. Participants were not informed about the reward-punishment schedule.

### Resting-state EEG

The Active-Two system (BioSemi, Amsterdam, The Netherlands) was used for recording the resting-state EEG. Thirty-two electrodes were placed and EEG data was sampled at 2,048 Hz and a default online low pass filter (DC to 400 Hz) was applied. Four minutes of resting-state EEG was recorded (2 min with the eyes open and 2 min with the eyes closed).

### Reward and punishment sensitivity

Carver and White’s (1994) orthogonally-dimensioned behavioral inhibition system (BIS) and behavioral activation system (BAS) self-report questionnaire was used to index the punishment and reward sensitivity of the subjects (van Honk et al., 2002). This questionnaire is derived from Gray’s framework of human personality (Gray, 1987), wherein BAS mediates approach behavior in response to cues of reward and BIS is sensitive to cues of punishment and activates avoidance.

### Procedure

Upon arrival at the laboratory, participants were informed about the experiment and written informed consent was obtained. Participants were seated in a dimly lit room and first filled in the BIS-BAS questionnaire. A subset (71) of the participants also filled in two other questionnaires that were part of another study. The cap and electrodes were placed and the resting-state EEG was recorded subsequently. Next, participants were subjected to the reversal learning gambling task after they received on-screen instructions. Participants were encouraged to win as much fictitious money as possible. Subjects chose the low or high value by pressing the left mouse button with their left thumb or by pressing the right mouse button with their right thumb, respectively. The duration of the task was approximately 20 min. Seventy-one participants were subjected to another task as part of larger study after completion of the reversal learning gambling task. Resting-state EEG was recorded in a separate session for this group of participants. Twenty subjects of this group were lost to follow-up after the first session. Therefore, no resting-state EEG was recorded for these subjects. Note that for all participants resting-state EEG was recorded before any task performance.

### Data reduction and statistical analyses

Percentage high risk-taking for each round was calculated and for each subject polynomial quadratic trend scores were computed for the percentage of high risk-taking across the six rounds by using the program DAR (Kenemans, 1991). These quadratic trend scores capture the U-shaped pattern of risk-taking which would be expected for individuals who successfully adapt behavior on the basis of shifts in reward-punishment contingencies (i.e., taking high risk during round 1 and 2, followed by low risk-taking during round 3 and 4, followed by high risk-taking during round 5 and 6). High positive quadratic trend scores represent good reversal learning. Note that this is essentially a regression procedure, as the individual readout measure is a coefficient representing the fit of the individual data to a quadratic (parabolic) model.

Next, we computed difference scores for the percentage high risk-taking representing the adjustment in behavior following a contingency reversal. This was computed for the reward (phase 1) to punishment (phase 2) transition (R-P), as follows: high risk phase 1 − % high risk phase 2. Difference scores for the punishment (phase 2) to reward (phase 3) transition (P-R) were computed as follows: % high risk phase 3 − % high risk phase 2

Subsequently, these difference scores were normalized for the individual’s total percentage risk-taking during the respective phases. These “reversal learning ratios” were calculated for the reward (phase 1) to punishment (phase 2) transition (R-P) and for the punishment (phase 2) to reward (phase 3) transition (P-R). R-P reversal learning ratio was calculated as follows:

$$ \frac{\%\kern0.5em  high\kern0.5em  risk\kern0.5em  phase\kern0.5em 1-\%\kern0.5em  high\kern0.5em  risk\kern0.5em  phase\kern0.5em 2}{\%\kern0.5em  high\kern0.5em  risk\kern0.5em  phase\kern0.5em 1+\%\kern0.5em  high\kern0.5em  risk\kern0.5em  phase\kern0.5em 2} $$

The P-R reversal learning ratio was computed as follows:

$$ \frac{\%\kern0.5em  high\kern0.5em  risk\kern0.5em  phase\kern0.5em 3-\%\kern0.5em  high\kern0.5em  risk\kern0.5em  phase\kern0.5em 2}{\%\kern0.5em  high\kern0.5em  risk\kern0.5em  phase\kern0.5em 3+\%\kern0.5em  high\kern0.5em  risk\kern0.5em  phase\kern0.5em 2} $$

Task performance (percentage high risk-taking) on the group level was investigated by testing the average of the individual quadratic trend scores against zero. In case of significance, follow-up paired-samples t-tests between successive rounds were conducted. A final paired-samples t-test was performed to test whether there was a difference between the first and the second reversal learning ratio on the group level.

Raw EEG signals were analyzed offline using Brain Vision Analyzer 2.0 (Brain Products GmbH). EEG data was resampled to 256 Hz and re-referenced to the average reference. Data were divided into 2-s segments which were baseline corrected in order to suppress potential DC drifts. An automatic artifact rejection procedure subsequently removed segments containing (ocular/muscle) activity exceeding 50 or -50 μV. On average, out of 120 segments, 114.2 (SD=1.5) segments remained for Fz, 118.1 (SD =3.7) remained for Cz and 112.4 (SD =11.4) remained for Pz. Spectral power in the theta (4–7 Hz), and beta (13–30 Hz) band was estimated by using a fast Fourier transformation (Hanning window: 10%). Spectral power estimates were averaged across segments and theta/beta EEG ratios were calculated for the midline electrodes Fz, Cz, and Pz (Schutter & van Honk, 2005; Schutter et al., 2006: Massar et al., 2014). Analyses for the eyes-open and eyes-closed condition separately demonstrated that theta/beta EEG ratio power values for the eyes-open and eyes-closed condition were highly correlated (rho =.821, p <.001). Data were therefore collapsed across both conditions.

Topographical plots were obtained for data as analyzed by the steps described above and for the same data re-referenced to the average mastoids instead of the average reference. The average of electrode P7/P8 was used as an approximation of the signal from the mastoids for 51 subjects for which we did not record from the mastoids. A 1–40 Hz band-pass filter and a subsequent eye blink correction (Gratton et al. method; Gratton, Coles, & Donchin, 1983) were applied to the data re-referenced to the mastoids (P7/P8) in order to ensure sufficient remaining data for the frontal channels. For both reference schemes we excluded individual electrode-channels in case less than half of the data segments (<60) were left for that channel after artifact rejection. The grand average of the spectral power for each channel was based on data of 75–108 subjects (mean ± SD, 100 ± 8.3) and 102–108 subjects (mean ± SD, 106 ± 1.9) for the average reference and the mastoids reference scheme, respectively.

**Fig. 2:**
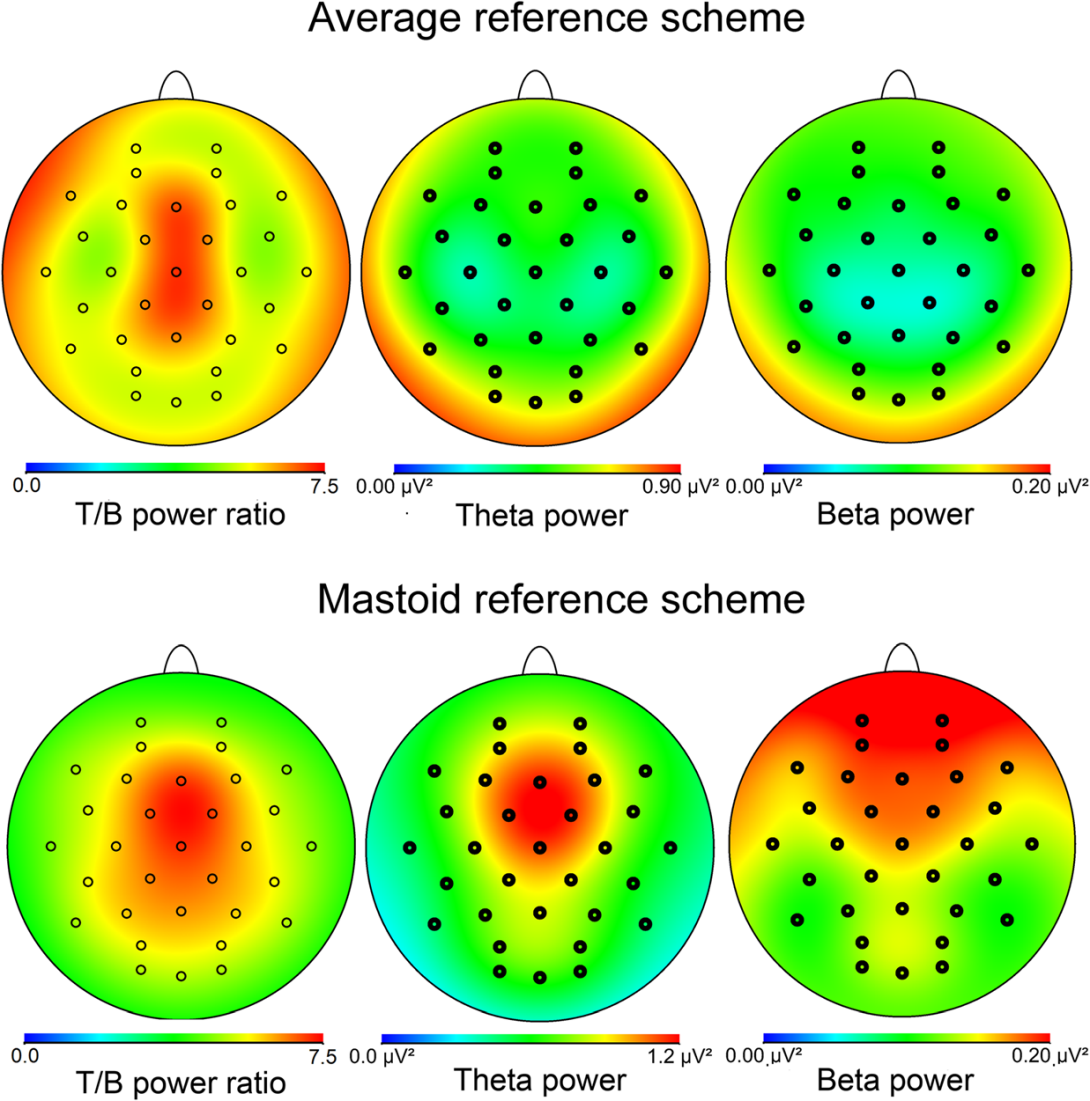
The topographical distribution of theta/beta EEG ratio, theta and beta power across the scalp. Theta/beta EEG ratio was maximal over midline frontal sites (Fz) (above, left). The other figures in the top row represent the topographical distribution of theta (middle) and beta power (right). Theta and beta power plotted separately were maximal over the parieto-occipital cortex when using the average reference. This pattern has also been found in a study by Cavanagh et al. (2012) and is a feature of the average reference. In contrast, the theta and beta power bands (as well as the theta/beta EEG ratio) showed a frontal maximum when the averaged mastoids were used as a reference (bottom row, middle and right figure, respectively). Note that the ratio between theta and beta power cancelled out the effects of the reference method on the scalp distribution of the power values

Table 1 displays average raw theta and beta power values for electrode Fz, Cz, and Pz. Figure 2 displays the topographical distribution of theta/beta EEG ratio, theta-, and beta power across the scalp. The top row represents data re-referenced to the average reference and shows that the distribution of the theta/beta EEG ratio was maximal over the mid-frontal cortex, as expected. Distributions of the average referenced theta and beta power separately were maximal over the parieto-occipital cortex. This pattern is a feature of the average reference scheme and has been observed before (Cavanagh et al., 2012). As expected, theta/beta EEG ratio and theta and beta power were all maximally distributed over the frontal cortex, surrounding electrode Fz, when the data were re-referenced to the average mastoids (Fig. 2, bottom row). Note that the theta/beta EEG ratio cancelled out the effects of the reference procedure on the scalp distribution.

**Table 1:** Mean theta and beta power in μV^2^

Electrode location	Theta Mean (SD)	Beta Mean (SD)
Fz	0.51 (0.23)	0.08 (0.05)
Cz	0.43 (0.22)	0.07 (0.05)
Pz	0.43 (0.26)	0.07 (0.04)

Significant Kolmogorov-Smirnov tests indicated that the distributions of the theta/beta EEG ratios at Fz, Cz and Pz deviated from normality. Therefore, Spearman’s correlations were used for the correlations involving theta/beta EEG ratios. A series of correlational analyses were conducted to test our main hypothesis that a high theta/beta EEG ratio measured at Fz is associated with poor reversal learning. We first examined the relationship between theta/beta EEG ratio at Fz and individual quadratic trend scores for the percentage risk-taking across the six rounds. Next, correlational analyses were conducted to test whether the difference scores (behavioral adaptations during the R-P and P-R transitions) were related to theta/beta EEG ratios. We subsequently investigated whether these latter correlations were contaminated by the subject’s overall percentage risk-taking by testing the correlation between theta/beta EEG ratios and the R-P and P-R reversal learning ratios. We additionally investigated the relationship between individual quadratic trend scores for the percentage risk-taking across the six rounds and theta/beta EEG ratios measured at Cz and Pz (for which we expected less strong associations in line with our prior studies). We also additionally explored the relationship between theta and beta power separately and reversal learning (i.e., quadratic trend scores).

Finally, Pearson’s correlations were conducted to test the relationship between reversal learning ratios and self-reported reward-punishment sensitivity (our second aim).

Alpha level was set to .05 for all analyses, unless stated otherwise. Bonferroni corrections were applied for follow-up paired samples t-tests. Greenhouse-Geisser adjustments were made when appropriate.

## Results

**Fig. 3:**
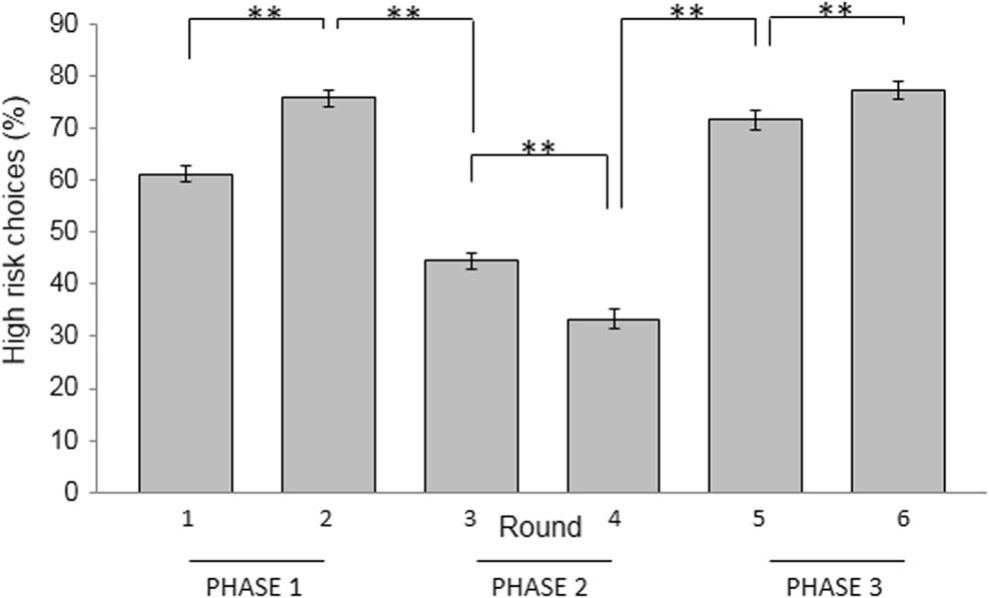
Percentage of high-risk choices during each round. Error bars represent ±1 SE. The quadratic trend for the percentage high risk-taking across the six rounds was highly significant (p < .001), indicating that, on average, participants learned to adapt their behavior

A significant quadratic trend for the percentage high risk-taking across the six rounds was found, F(1,127) = 152.7, p < .001, which confirmed that participants learned to successfully adapt their decision making. Follow-up paired-samples t-tests showed significant increases in high risk-taking in the first phase (round 1 and 2) of the task, t(127) = -10.49, p < .001. Furthermore, a significant decrease in high risk-taking was observed between round 2 and round 3, t(127) = 12.35, p < .001. During phase 2 (round 3 and 4) a further decline in high risk-taking was found, t(127) = 6.66, p < .001, while risk-taking increased again between round 4 and round 5, t(127) = -14.59, p < .001, and during phase 3 (round 5 and 6), t(127) = -4.08, p < .001. The significant changes in high risk decision making between round 2 and 3 and between round 4 and 5 further demonstrate that participants learned to adapt behavior on the basis of shifts in reward-punishment contingencies. Figure 3 shows the percentage risk-taking during each round and reversal learning across the task. Note that all p-values were below the Bonferroni-corrected threshold for significance of p = .01.

**Fig. 4:**
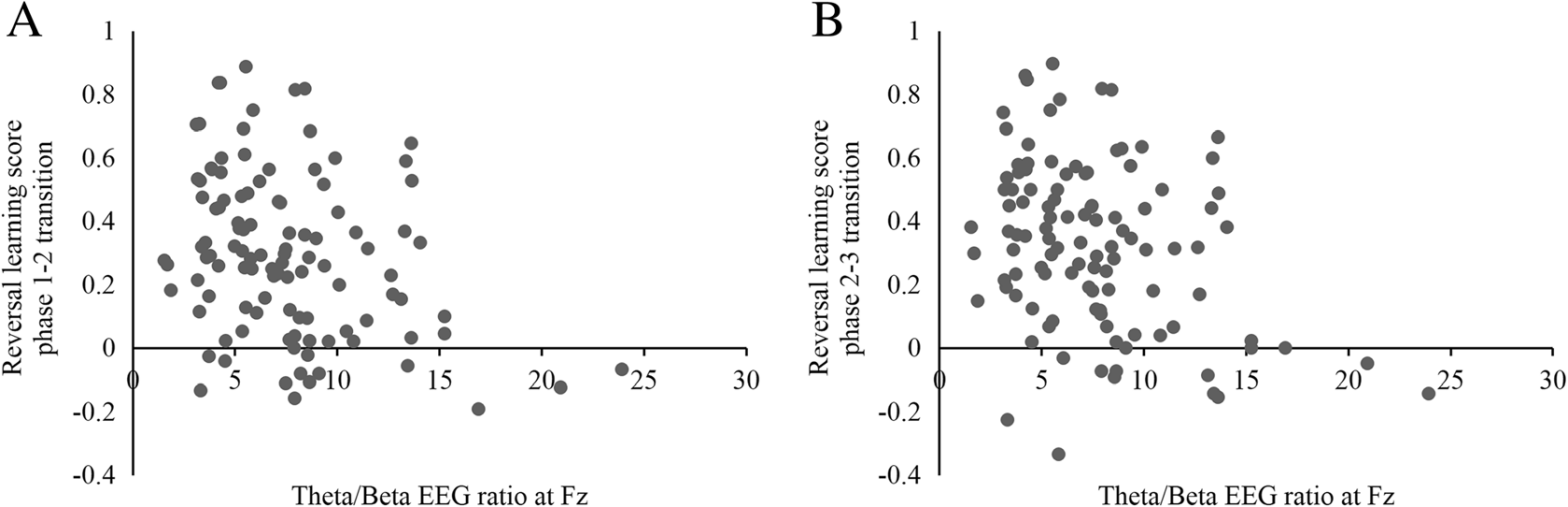
Reversal learning scores correlate negatively with theta/beta EEG ratio. A reversal learning score of 1 represents perfect learning. Scatterplots are shown for reversal learning during the phase 1–2 transition (**panel A**) and 2–3 transition (**panel B**)

A significant negative correlation was observed between theta/beta EEG ratio measured at Fz and the individual quadratic trend scores for the percentage high risk-taking across the rounds (a high positive score means good learning), rho = -.306, p = .001. These results were confirmed by significant negative correlations between theta/beta EEG ratio measured at Fz and percentage high risk difference scores (behavioral adaptations after a contingency reversal), rho = -.295, p=.002 (phase 1-2) and rho = -.286, p = .003 (phase 2-3). These correlations remained significant when the normalized difference scores (i.e., reversal learning ratios) were used instead, rho = -.285, p = .003 (phase 1–2 transition) and rho = -.277, p = .004 (phase 2–3 transition). Together these results indicate that participants with high theta/beta EEG ratio were less inclined to change to more adaptive decision making when reward-punishment schedules were reversed. See Fig. 4A (phase 1–2 transition) and 4B (phase 2–3 transition).

In addition, a paired samples t-test comparing reversal learning ratios between the first and the second reward-punishment transition revealed significantly larger reversal learning ratios for the second transition, mean ± SD = 0.32 ± 0.27, compared to the first, mean ± SD= 0.29 ± 0.25, t(127) = -3.27, p = .001.

We also tested whether theta/beta EEG ratio measured at electrode Cz and Pz similarly predict reversal learning. Significant negative correlations between theta/beta EEG ratio and individual quadratic trend scores for the percentage risk-taking were also observed for the electrodes Cz and Pz, rho = -.243, p = .011, and rho = -.316, p = .001, respectively.

**Fig. 5:**
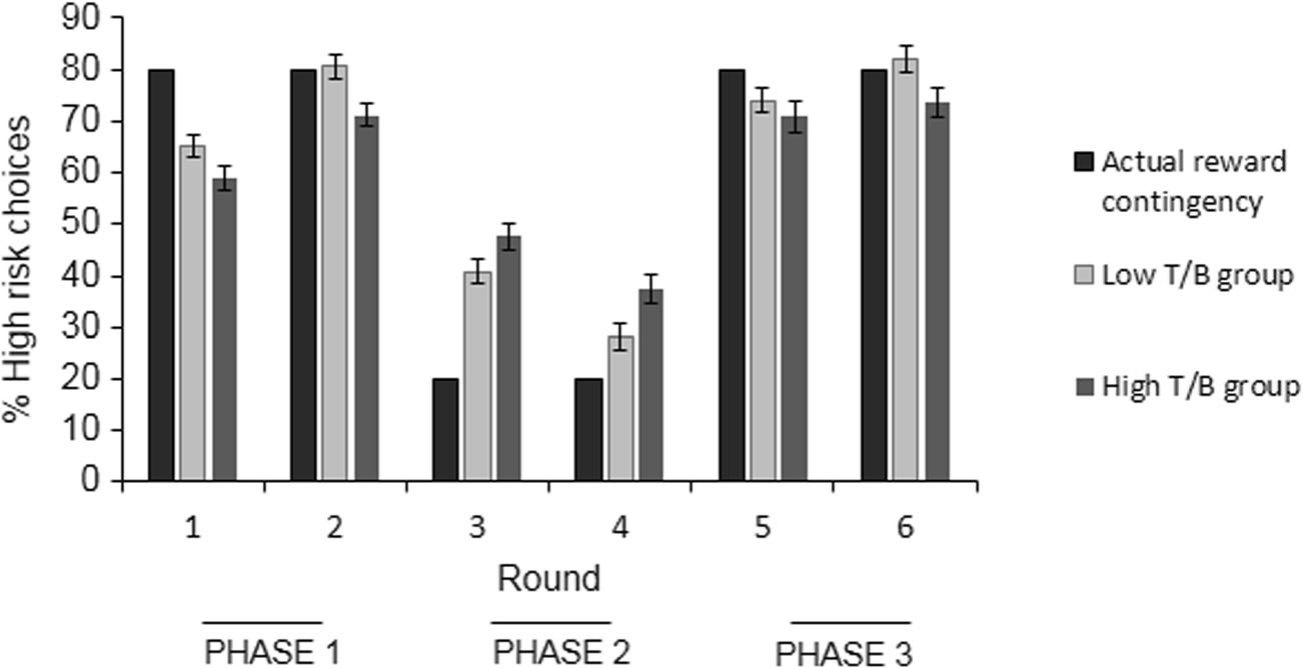
Percentage of high-risk choices during each round for the high and low theta/beta EEG ratio group. Participants were categorized as having either a low (light grey bars) or a high (dark grey bars) theta/beta EEG ratio, based on a median-split. The figure represents the percentage high risk choices during each of the six rounds for both groups separately, relative to the actual reward contingency during each round (black bars). Error bars represent ±1 SE

Next, we examined whether the relationship between theta/beta EEG ratio at Fz and reversal learning was explained by risky decision making during phase 2 or by low risk-taking during phases 1 and 3. Therefore, correlations were computed between theta/beta EEG ratio at Fz and the percentage risk-taking during each of the three phases. Interestingly, this analysis revealed that individuals with a high theta/beta EEG ratio made less risky decisions during both reward phases, rho = - .253, p = .008 (phase 1), rho = - .213, p = .027 (phase 3). There was also a significant positive correlation between theta/beta EEG ratio and percentage risk-taking during phase 2, rho = .216, p = .025. Figure 5 displays the percentage risk taking during each of the six rounds for participants with a low (below the median) and high (above the median) theta/beta EEG ratio separately. Percentage high risk-taking in phase 1 and 3 was furthermore found to correlate negatively with percentage high risk-taking in phase 2, rho = -.199, p = .024; rho = -.189, p = .033, respectively, indicating that individuals exhibiting low risky behavior in phase 1 and 3 are for a large part the same individuals exhibiting high risky behavior in phase 2. These correlations were non-significant (p values > .116) when controlling for theta/beta EEG ratio.

A significant positive correlation between theta and beta power was found, rho = .516, p < .001. However, neither of these measures correlated with the individual quadratic trend scores for the percentage high risk-taking across the rounds, rho = -.056, p = .564 (theta power) and rho = .174, p = .07 (beta power). Results of the Steiger tests (1980) indicated that the strength of the latter correlations, and the correlation between theta/beta EEG ratio and the quadratic trend scores were significantly different; Z = 4.55, p < .01 (theta/beta EEG ratio versus theta power) and Z = 3.62, p < .01 (theta/beta EEG ratio versus beta power). These findings show that the theta/beta EEG ratio explains unique variance in reversal learning.

No significant correlations were observed between self-reported reward and punishment sensitivity and reversal learning ratios (p values > .4). However, a significant negative correlation between self-reported punishment sensitivity and theta/beta EEG ratio was observed, rho = -.211, p = .028. Finally, the correlation between theta/beta EEG ratio and self-reported reward sensitivity was not significant (p = .883).

## Discussion

The primary aim of the current study was to investigate whether the theta/beta EEG ratio is associated with reversal learning in an environment with changing reward-punishment contingencies. Results showed that participants with a high theta/beta EEG ratio were less able to adapt to the change in reward-punishment contingency. The negative correlation between theta/beta EEG ratio on the one hand and reversal learning on the other hand is consistent with our predictions. In line with the findings by Schutter and colleagues (2005), we theorized that the theta/beta EEG ratio reflects the inverse of cortical regulating signals relative to subcortical to-be-regulated activity (reward drive). Theta/beta EEG ratio was associated with risky decision-making in our previous studies, and the latter was hypothesized to be negatively related to the ability to adapt to changing choice-reward/punishment contingencies. Hence, participants with high theta/beta EEG ratio would be less able to flexibly switch between decision-making strategies during the task and less able to adjust behavior. Reversal learning in an environment in which previously rewarded actions suddenly have opposite outcomes requires that individuals switch between exploitation of known responses and exploration of alternatives (Daw et al., 2006). The results on the current task showed that, on average, individuals were able to adapt their behavior both within and between the reward-punishment contingency phases of the reversal learning task. However, individuals with relatively high theta/beta EEG ratio were less able to do so.

Notably, participants with high theta/beta EEG ratio made = in fact less risky decisions when high risk-taking was rewarding. At first sight this finding seems at odds with evidence from prior studies that supports a relationship between increased theta/beta EEG ratio and high risk-taking and approach behavior (Massar et al., 2014; Schutter & van Honk, 2005). Results from these studies show that subjects with a high theta/beta EEG ratio keep choosing from high-risk decks during the IGT, while low theta/beta EEG ratio subjects gradually learn which deck is most beneficial and adapt their choices accordingly.

The present results suggest that theta/beta EEG ratio specifically reflects the ability to adapt choices in response to changing contingencies, and that this may be what drives the association between theta/beta EEG ratio and optimal performance also in the traditional implementation of the IGT. In accordance with our findings, a recent double-blind randomized controlled study applied 5-Hz transcranial alternating current stimulation (tACS) to the frontal cortex, which improved reversal learning in healthy volunteers. Results showed that even though volunteers improved on learning ability, they were less inclined to actually change their risk taking accordingly. Notably, EEG recordings showed a significant lowering of spontaneous theta/beta EEG ratios (Wischnewski, Zerr, & Schutter, 2016).

A large body of empirical work has shown that mid-frontally generated theta oscillations are elicited during signals of punishment and conflict (Cavanagh, Figueroa, Cohen & Frank, 2011; Cavanagh & Frank, 2014; Cavanagh & Shackman, 2015; Cohen, Ridderinkhof, Haupt, Elger, & Fell, 2008). More specifically, mid-frontal theta oscillations are thought to signal reward prediction error signals that originate from the subcortical dopamine system and are elicited when outcomes are worse than expected (Holroyd and Coles, 2002). Cavanagh and colleagues (2011) also showed that theta activity was enhanced when participants were uncertain about their responses during a probabilistic reward task. These theta signals during uncertainty seem particularly pronounced in high trait-anxious individuals (Cavanagh & Shackman, 2015) and are thought to signal the need for cognitive control (Cavanagh & Frank, 2014) or behavioral adaptation (Cavanagh, Frank, Klein, & Allen, 2010).

It should, however, be noted that these relations pertain to task-related or induced theta signals. These studies may suggest that task-related theta signals are associated with better reversal learning. The present results, however, demonstrate that high spontaneous theta/beta EEG ratio is associated with less ability for adaption after a reversal. One explanation for this apparent discrepancy is that high spontaneous theta activity is associated with a reduced theta response to task demands or stimuli that prompt adaptation. Even though one study found an inverse relation between pre-stimulus theta power and stimulus-induced theta power (Klimesch et al., 2004), to the best of our knowledge there are no studies available that have directly addressed this issue. Note that pre-stimulus theta activity within a task context cannot be equated with resting-state spontaneous theta activity. Another clue was more recently provided by Massar et al. (Massar, Rossi, Schutter, & Kenemans, 2012). These authors reported a negative correlation between resting-state theta activity and the feedback-related negativity (FRN) to negative feedback stimuli, albeit only in individuals with high punishment sensitivity. The FRN is generally considered to be an evoked-theta-dominated response (Cavanagh et al., 2012), and it overlaps with induced theta activity with respect to the association with reversal-learning aspects (Mas-Herrero & Marco-Pallarés, 2014). Yet, a direct comparison between resting-state theta activity and task-related theta responses has not been made. Note that we also found a negative correlation between theta/beta EEG ratio and self-reported punishment sensitivity. Conceptually, this relation is also consistent with the idea of a negative relation between resting-state theta (/beta) activity and feedback-induced theta oscillations (e.g., FRN elicited by punishment).

As already mentioned, participants with a high theta/beta EEG ratio showed a pattern of low risk-taking during phase 1 and 3 of the current task. High risk-taking was rewarded in 80% of the cases during these phases. However, still 20% of the high-risk choices led to a loss of money. Apparently, in high theta/beta EEG ratio individuals, high risk loss may have promptly resulted in low risk taking, whereas it would have been more adaptive to use reward and punishment feedback information over a larger number of trials. This could indicate that endogenous high theta activity correlates with increased prediction errors after high-risk losses (i.e., under uncertainty). This would in fact predict that endogenous high theta relative to beta activity is associated with larger negative-feedback-induced theta oscillations, which is contrary to the notion presented in the previous paragraph. This apparent contradiction should be addressed in future research including assessment of feedback-induced theta in reversal-learning gambling contexts.

In addition, endogenous low beta relative to theta activity may be associated with a lack of prefrontal cortical control over decision-making strategies (exploit vs. explore) during the task. Decreased cognitive flexibility (i.e., low beta activity) together with uncertainty and large prediction errors after high-risk losses (i.e., high theta activity) may have caused a delay in learning reward-punishment contingencies in participants with high theta/beta EEG ratio.

Furthermore, the correlation between theta/beta EEG ratio and the quadratic reversal learning pattern explained significantly more variance than the correlation for theta and beta power separately. These latter tests revealed no significant results. This finding indicates that theta/beta EEG ratio explains unique variance in reversal learning. However, since the separate tests for theta and beta power were not part of our original hypothesis, these findings should be replicated in an independent sample.

Although it was expected that reversal learning would be predominantly associated with theta/beta EEG ratio recorded at the frontal electrode (Schutter et al., 2006: Massar et al., 2014), the current results indicate that the central and parietal theta/beta EEG ratio also predicts reversal learning. This finding concurs with a prior study in which a relationship was observed between disadvantageous decision making and theta/beta EEG ratio measured at the mid-frontal (Fz) and parietal sites (Pz) (Schutter & van Honk, 2005).

A second aim of the present study was to explore whether self-report measures of reward and punishment sensitivity predict reversal learning during the RLG task. The expected inverse correlation between reward sensitivity (BAS) and reversal learning, and the positive correlation between punishment sensitivity (BIS) and reversal learning were not found. We did, however, observe a significant negative relationship between self-reported punishment sensitivity and theta/beta EEG ratio.

Our study leaves open a number of issues for further investigation. First, our design cannot differentiate between learning the reward-punishment contingency and executing the correct strategy. Second, our results raise the issue of alternative reward-punishment contingencies for high-risk choices (e.g., R:P 60:40 and 40:60%) on reversal learning and its relationship with theta/beta EEG ratio. Third, the RLG task always started with a phase during which 80% of the high-risk choices were rewarded (R:P 80:20). This leaves open the question of whether starting with the alternative R:P contingency phase (i.e., 80% punishment for high-risk choices) would yield comparable results. Context-related differences such as task offset may be important to investigate given that, for example, patients with orbitofrontal cortex damage show reduced learning during the IGT compared to controls, but *only* when the first cards of the high-risk deck consist of wins (Fellows, 2007). Fourth, a relation between risk taking and theta asymmetry rather than overall power has also been reported (e.g., Studer, Pedroni, & Rieskamp, 2013), prompting the question if there are similar relations between risk taking and/or reversal learning and theta/beta EEG ratio asymmetry.

In conclusion, one’s ability to adapt to changing reward-punishment environments by adjusting behavior on the basis of shifts in emotional significance is vital for behavioral flexibility in changing environments (Clark et al., 2004). The present study demonstrates that individuals with an increased ratio between low frequent oscillations in the theta range and high frequent oscillations in the beta range during resting state exhibit lower levels of behavioral flexibility. This was reflected by a reduced ability to respond adaptively and to adjust behavior after a reversal of reward contingencies. Higher levels of theta/beta EEG ratios were associated with poorer reversal learning, which is arguably due to a decreased ability to learn which choice was more likely to yield a reward.
